# Multiple introductions of divergent lineages and admixture conferred the high invasiveness in a widespread weed (*Hypochaeris radicata*)

**DOI:** 10.1111/eva.13740

**Published:** 2024-06-21

**Authors:** Soo‐Rang Lee, Tae‐Young Choi, Dong‐Chan Son

**Affiliations:** ^1^ Department of Biology Education, College of Education Chosun University Gwangju South Korea; ^2^ Division of Forest Biodiversity and Herbarium Korea National Arboretum Pocheon Korea

**Keywords:** demographic history, *Hypochaeris radicata*, invasive species, multiple introductions, population bottleneck, range expansion

## Abstract

Biological invasion consists of spatially and temporally varying stages, accompanied by ecological and evolutionary changes. Understanding the genomics underlying invasion dynamics provides critical insights into the geographic sources and genetic diversity, contributing to successful invasions across space and time. Here, we used genomic data and model‐based approaches to characterize the invasion dynamics of *Hypochaeris radicata* L., a noxious weed in Korea. Genetic diversity and assignment patterns were investigated using 3563 SNPs of 283 individuals sampled from 22 populations. We employed a coalescent‐based simulation method to estimate demographic changes for each population and inferred colonization history using both phylogenetic and population genetic model‐based approaches. Our data suggest that *H. radicata* has been repeatedly been introduced to Korea from multiple genetic sources within the last 50 years, experiencing weak population bottlenecks followed by subsequent population expansions. These findings highlight the potential for further range expansion, particularly in the presence of human‐mediated dispersal. Our study represents the first population‐level genomic research documenting the invasion dynamics of the successful worldwide invader, *H. radicata*, outside of Europe.

## 1 INTRODUCTION

Biological invasions have caused global change with harmful impacts on biodiversity, human health, and agriculture over the past few centuries (Sax et al., 2005). The invasion process takes place in a series of consecutive stages, including transport, introduction, population establishment, and spread (Theoharides & Dukes, 2007), although the stages are often not discrete. Invasion success largely depends on how varying environmental filters and eco‐evolutionary factors act at each stage. Many theoretical models have proposed how various factors shape each stage of invasion, such as increased success in the initial stages due to high propagule pressure and human impact (Blackburn et al., 2020; Dlugosch et al., 2015; Gioria et al., 2023; Sherpa & Després, 2021; Simberloff, 2009). However, the factors shaping later stages of population establishment and spread remain less elucidated, but investigating them is crucial for understanding the progression of invasions.

Population establishment and landscape spread are complex processes associated with multiple factors such as spatial and demographic shifts, long‐distance dispersal, species traits, landscape heterogeneity, and human impacts (Gioria et al., 2023; Theoharides & Dukes, 2007). Observing this process requires extensive sampling of local populations at different time scales across varying landscapes. Alternatively, patterns of population establishment and spread can be indirectly inferred from the genetic data of extant populations (Colautti et al., 2006; Estoup & Guillemaud, 2010; Sherpa & Després, 2021; Welles & Dlugosch, 2019). For example, genomic data analysis (2696 SNPs) of 24 burcucumber populations revealed recent bottlenecks and repeated introductions from multiple and genetically divergent sources during colonization (Lee & Son, 2022).

Genetic inferences, however, are not straightforward and are often confounded by the complex demographic changes, particularly for colonizing species (Dlugosch et al., 2015; Estoup & Guillemaud, 2010). Alien species can originate from a small number of propagules introduced from one or a few sources in their native regions, consequently experiencing the founder effects (Dlugosch et al., 2015). Another notable feature of biological invasion is short divergence times (Estoup & Guillemaud, 2010; Welles & Dlugosch, 2019). During an invasion, populations may rapidly spread from the original founding foci and subsequent leading‐edge populations. This rapid expansion likely allows limited time for genetic variations to accumulate. The paucity of divergence time often leads to lower levels of population differentiation. Consequently, the low divergence can reduce the power to infer demographic history. Population bottlenecks, on the other hand, will increase the intensity of genetic drift, resulting in elevated population differentiation (Dlugosch & Parker, 2008b), improving, genetic inference of population history (Estoup & Guillemaud, 2010).

**FIGURE 1:**
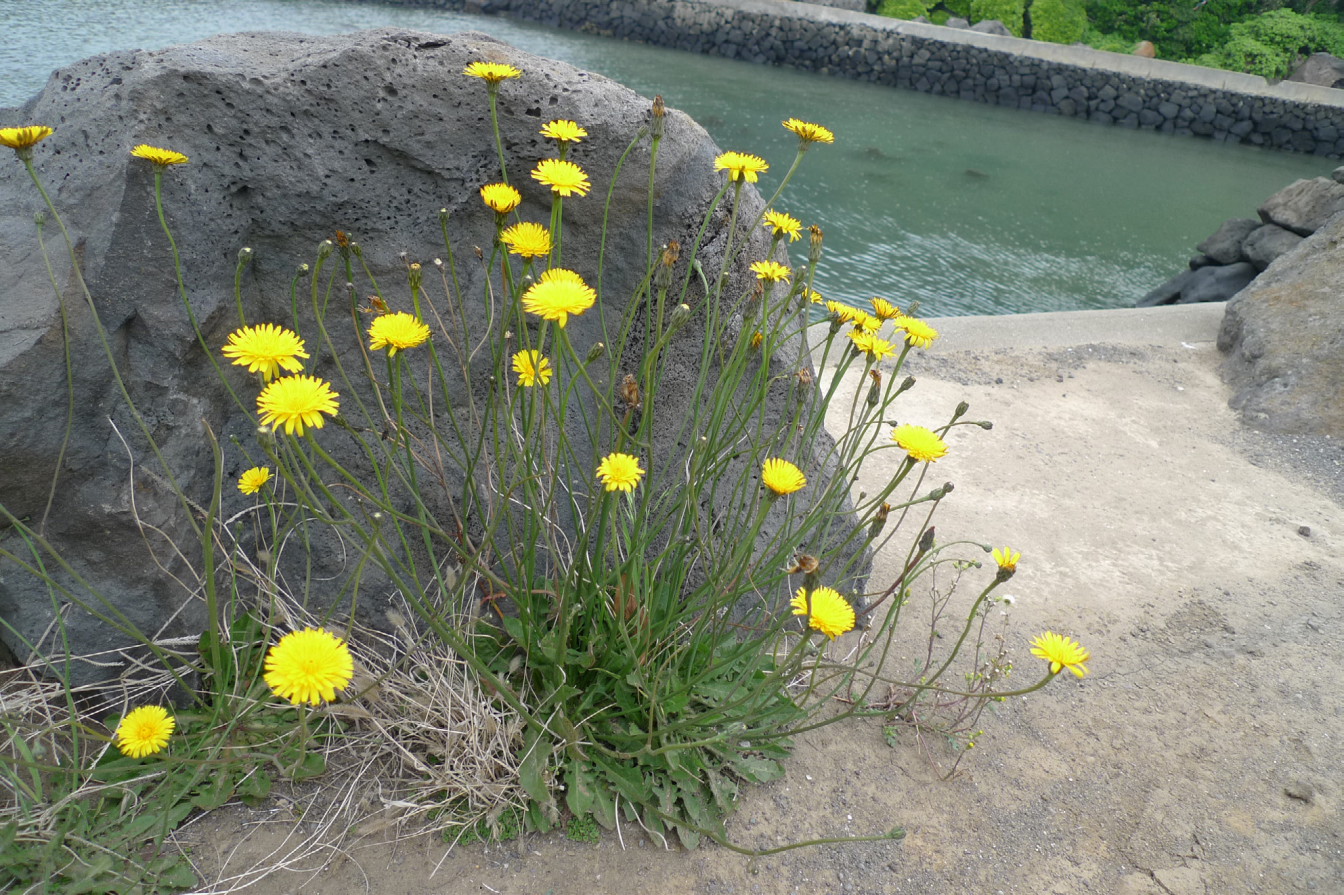
*Hypochaeris radicata* growing on Jeju Island. The picture was photographed by Sa‐Bum Jang in April 2012.

*Hypochaeris radicata* L. (Asteraceae; 2*n* = 8; native to Mediterranean region; Figure 1), is an herbaceous perennial distributed throughout nearly all continents (Cameron, 1988; Turkington & Aarssen, 1983; Weiss et al., 2003). The species grows most vigorously in well‐drained soils and open spaces such as roadsides, pastures, cultivated fields, lawns, and wastelands (Cameron, 1988; Turkington & Aarssen, 1983). Unsurprisingly, given its wide distribution in its native range (from Scandinavia to the Alps), the plant exhibits significant phenotypic and physiological plasticity (Cameron, 1988; Turkington & Aarssen, 1983), a trait crucial for its high invasiveness (Baker, 1974). *Hypochaeris radicata* is drought‐tolerant with a deep‐rooted system (Cameron, 1988) and readily adapts to a variety of climatic conditions, except for extreme winter cold (Turkington & Aarssen, 1983). *Hypochaeris radicata* has become a highly successful invasive weed in Northern and Central Europe, Australia, East Asia, and South America (Cabrera, 1987; Dellow et al., 2002; Doi et al., 2006; Turkington & Aarssen, 1983). Its range in Australia and Japan has recently expanded and is expected to continue growing in temperate regions (Dellow et al., 2002; Doi et al., 2006; Ortiz et al., 2008; Takematsu & Ichizen, 1987). Currently, the species ranks among the most abundant plants in temperate perennial pastures in Australia and Japan (Dellow et al., 2002; Doi et al., 2006).

Besides high tolerance to environmental stresses, *H. radicata* is endowed with a suit of attributes to be an ideal weed (Baker, 1974). *Hypochaeris radicata* is pollinated by a number of insects and produces a large amount of minute seeds (ca. 2400 achenes per plant, each weighed 0.6–0.8 mg; Ho, 1964) for nearly the entire growing season (from late spring to early fall; Cameron, 1988). The seeds can easily disperse by wind traveling up to 2 m (Turkington & Aarssen, 1983). Dormancy or specific environmental conditions are not required for seed germination, and the seed viability remains high over 7 months (Cameron, 1988; Doi et al., 2006). The species exhibits high competitiveness, exerting allelopathic effects on other plants and rapidly covering an area through its rosette behavior (Turkington & Aarssen, 1983).

In Korea, the first official record of *H. radicata* was documented in 1988 by a botanist, and subsequently, the plant was observed in parks, golf courses, and ski fields (Ahn et al., 2001). The species was published as a newly colonized alien plant in Korea by Sun et al. (1992). Prior to its official documentation, local citizens had already noted the presence of *H. radicata*, particularly around the pastures on Jeju Island, even before the 1980s (Ahn et al., 2001). This observation led to the hypothesis that the species might have been introduced to Jeju island from Europe and/or America as seeds along with the grasses for cattle grazing (Ahn et al., 2001), similar to its introduction in Japan much earlier (Sun et al., 1992). However, the timing of the introduction and the colonization routes remain largely unknown. Currently, *H. radicata* is one of the primary target species for control since it was designated as a harmful invasive species (only 17 invasive plants were designated) by the Ministry of Environments in Korea. The species is abundant along roadsides, near agricultural fields, and in disturbed areas with open spaces. Particularly on Jeju island, the plant occupies nearly all open spaces, posing a great threat to biodiversity and ecosystem integrity (NIER, 2006). In fact, quadrat surveys have demonstrated a reduction in species diversity at sites where *H. radicata* is present, likely attributed to its allelopathic effects and high‐reproductive rates (NIER, 2006). Despite its detrimental effects on natural vegetation and the high potential for further range expansion, the ecological and evolutionary understanding of *H. radicata* is largely lacking.

In the present study, we employed genomic data and model‐based approaches to characterize the invasion dynamics of *H. radicata* in Korea. Considering the current distribution pattern with high density on Jeju island, we hypothesized that the species was initially introduced to Jeju and subsequently spread to inland areas. Severe population bottlenecks were also postulated, given the recent introduction and rapid expansion history. To test our predictions, we first assessed genetic diversity and spatial assignment patterns to discern the patterns of population divergence in Korea. We then quantified demographic changes for each population by estimating contemporary and past effective population sizes with a coalescent‐based simulation method. Last, we inferred the demographic history of the invasion using both phylogenetic and model‐based approaches.

## 2 MATERIALS AND METHODS

### 2.1 Study system, sample collection, and DNA extraction

#### 2.1.1 Study system

*Hypochaeris radicata* is a self‐incompatible and outcrossing species. However, the species may vegetatively propagate through perennating buds (Turkington & Aarssen, 1983). The plant forms a basal rosette of lobed leaves, around 15 cm in diameter, with a fleshy taproot. Additionally, it produces several flowering stalks that can reach heights of 40–50 cm and often branch (Picó et al., 2004).

#### 2.1.2 Sample collection

**FIGURE 2:**
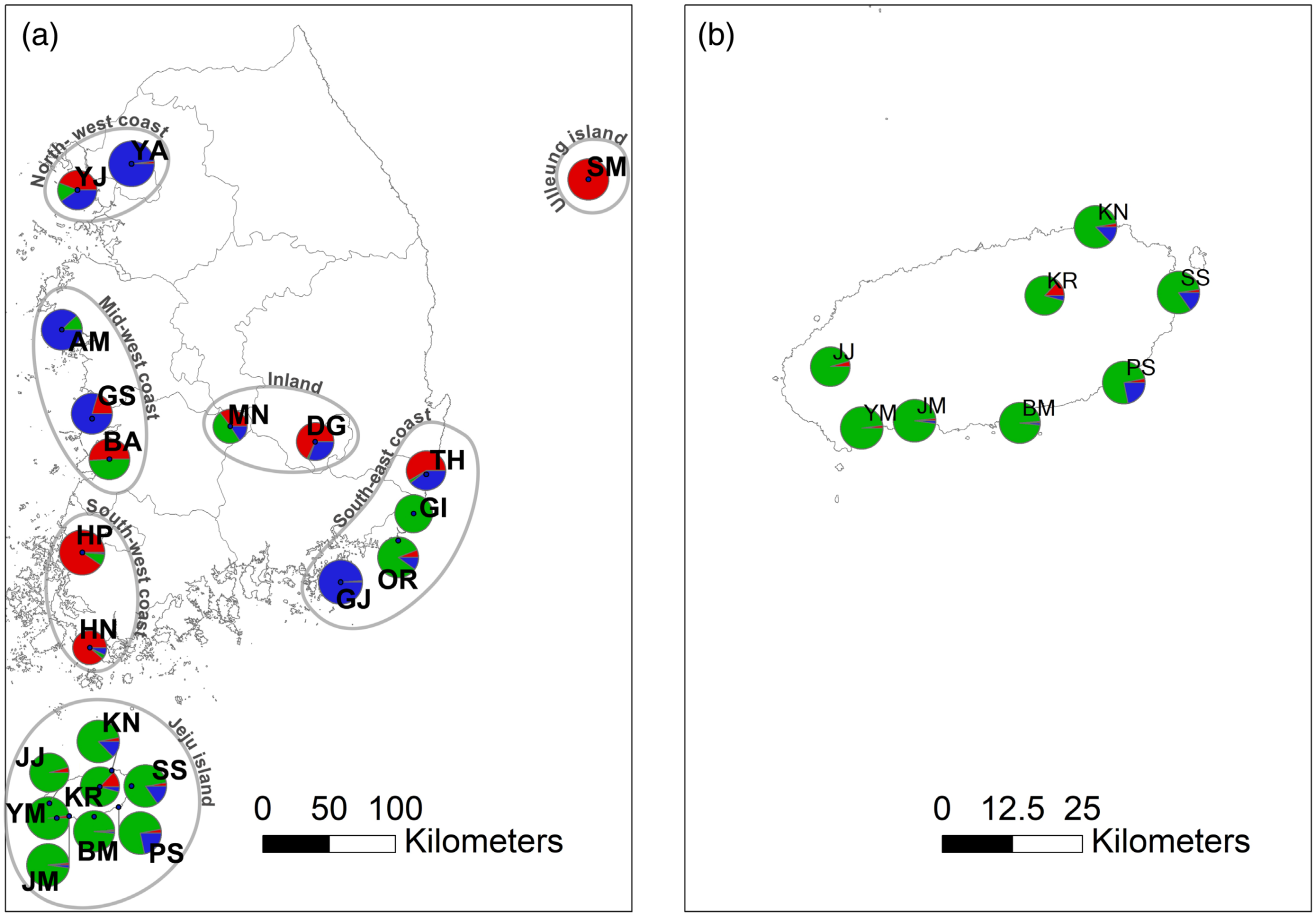
Bayesian assignment pie charts (*K* = 3) on a map of 22 *Hypochaeris radicata* populations sampled in Korea. (a) All sampled populations. (b) Fine‐scale map of 8 sampled populations from Jeju island. Colors depict the assignment of loci into 3 estimated groups. See Table 1 for population acronyms. The sizes of the pie charts are proportional to the relative sample sizes of each population.

We collected leaf tissue of 283 individuals from 22 populations in the summer of 2022 (Table 1; Figure 2). Except for Jeju island, the sampled populations were randomly selected across the Korean peninsula. The sampled populations were dispersed at ~50 km intervals. Because the plant is widely distributed on Jeju island with high density, we hypothesized that the island served as the source of the species’ spread in Korea. Therefore, we collected samples at closer intervals across Jeju island (eight populations collected within 30 km radius). Except for Jeju island, the sampled populations were randomly selected across the whole Korean peninsula. The sampled populations were dispersed at ~50 km intervals. We defined a population as a group of plants inhabiting an area within a few hundred meters radius, taking into account the dispersal mode and growth habit of the species (Turkington & Aarssen, 1983). Most populations collected were in maritime habitats with sandy soil except for four populations DG, HP, MN, and YA (Figure 2). Of the four, three populations were located alongside paved roads, while MN was placed in a national park near the service roads (Mt. Deokyu). Although the plant primarily propagates vegetatively through perennating buds (Turkington & Aarssen, 1983), we made efforts to avoid collecting multiple samples from a single plant or close relatives by distancing at least 10 m between sampled individuals. We stored the leaf tissues at room temperature in plastic Ziplock bags sealed with silica gel desiccant until further use.

**TABLE 1:** Summary of genetic diversity parameters and collection sites for *Hypochaeris radicata* in Korea.

Location	Population acronym	Assigned group	No. of individuals	GPS coordinates		He	Ho	Na
				*X*	*Y*			
Anmyeon‐eup, Taean‐gun, Chungcheongnam‐do	AM	pop3	13	36.54940	126.32945	0.234 ± [0.003]	0.191 ± [0.003]	1.68 ± [0.006]
Jusan‐myeon, Buan‐gun, Jeollabuk‐do	BA	–	10	35.67233	126.73409	0.176 ± [0.003]	0.166 ± [0.003]	1.77 ± [0.008]
Hahyo‐dong, Seogwipo‐si	BM	pop1	13	33.24210	126.61653	0.199 ± [0.003]	0.157 ± [0.003]	1.62 ± [0.006]
Okpo‐myeon, Dalseong‐gun, Daegu‐gwangyeogsi	DG	pop2	13	35.78336	128.44771	0.205 ± [0.003]	0.170 ± [0.003]	1.62 ± [0.007]
Ilgwang‐myeon, Gijang‐gun, Busan‐gwangyeogsi	GI	pop1	11	35.28184	129.25509	0.231 ± [0.003]	0.194 ± [0.003]	1.77 ± [0.006]
Dongbu‐myeon, Geoje‐si, Gyeongsangnam‐do	GJ	pop3	15	34.82641	128.64122	0.251 ± [0.003]	0.199 ± [0.003]	1.71 ± [0.005]
Osikdo‐dong, Gunsan‐si, Jeollabuk‐do	GS	pop3	13	35.94804	126.58585	0.238 ± [0.003]	0.195 ± [0.003]	1.67 ± [0.006]
Songji‐myeon, Haenam‐gun, Jeollanam‐do	HN	pop2	10	34.39144	126.57635	0.198 ± [0.003]	0.158 ± [0.003]	1.86 ± [0.007]
Eomda‐myeon, Hampyeong‐gun, Jeollanam‐do	HP	pop2	16	35.03782	126.50998	0.244 ± [0.003]	0.181 ± [0.002]	1.7 ± [0.005]
Hangyeong‐myeon, Jeju‐si	JJ	pop1	12	33.33086	126.25331	0.185 ± [0.003]	0.155 ± [0.003]	1.77 ± [0.007]
Yerae‐dong, Seogwipo‐si (Jungmoon)	JM	pop1	14	33.24503	126.41484	0.172 ± [0.003]	0.159 ± [0.003]	1.78 ± [0.006]
Gujwa‐eup, Jeju‐si	KN	–	14	33.55739	126.76037	0.216 ± [0.003]	0.159 ± [0.003]	1.67 ± [0.006]
Jochon‐eup, Jeju‐si	KR	–	12	33.44690	126.66300	0.221 ± [0.003]	0.165 ± [0.003]	1.7 ± [0.006]
Seolcheon‐myeon, Muju‐gun, Jeollabuk‐do (Mt Deokyu)	MN	–	9	35.89400	127.74064	0.205 ± [0.003]	0.184 ± [0.003]	1.64 ± [0.007]
Oryukdo‐dong, Busan‐gwangyeogsi	OR	pop1	13	35.10161	129.12215	0.235 ± [0.003]	0.186 ± [0.003]	1.68 ± [0.006]
Pyoseon‐myeon, Seogwipo‐si	PS	–	14	33.30743	126.81503	0.206 ± [0.003]	0.152 ± [0.002]	1.66 ± [0.006]
Seo‐myeon, Ulleung‐gun, Gyeongsangbuk‐do	SM	pop2	13	37.51064	130.80887	0.166 ± [0.003]	0.147 ± [0.003]	1.53 ± [0.008]
Seongsan‐eup, Seogwipo‐si	SS	–	14	33.45213	126.91939	0.207 ± [0.003]	0.135 ± [0.002]	1.64 ± [0.006]
Myeongchon‐dong, Ulsan‐gwangyeogsi (Taehwa river)	TH	pop2	12	35.54603	129.36548	0.237 ± [0.003]	0.195 ± [0.003]	1.69 ± [0.006]
Jangheung‐myeon, Yangju‐si, Gyeonggi‐do	YA	pop3	16	37.67773	126.91516	0.201 ± [0.003]	0.142 ± [0.003]	1.6 ± [0.007]
Unseo‐dong, Jung‐gu, Incheon‐gwangyeogsi	YJ	–	12	37.49875	126.45378	0.210 ± [0.003]	0.186 ± [0.003]	1.63 ± [0.007]
Andeok‐myeon, Seogwipo‐si	YM	–	14	33.23278	126.31396	0.199 ± [0.003]	0.146 ± [0.002]	1.64 ± [0.006]

*Note*: Assigned groups refer to the redefined groups for ABC analysis. He is expected heterozygosity. Ho is observed heterozygosity and Na is the number of alleles adjusted by the population sample sizes. The numbers in brackets are the standard errors.

#### 2.1.3 DNA extraction

Genomic DNA was extracted from the dried leaf tissues using the DNeasy Plant Mini Kit (Qiagen, Hilden, Germany) following the manufacturer's protocol. Extracted DNA was visualized on a 1% agarose gel through gel electrophoresis for quality check. The quantity of DNA was measured on a Qubit 4 Fluorometer (Thermo Fisher Scientific, MA), and finally the isolated DNA was stored at −20°C.

### 2.2 Restriction site‐associated DNA (RAD)‐library preparation & genotyping

#### 2.2.1 Library preparation and sequencing

We genotyped the collected samples using the 3RAD (Bayona‐Vásquez et al., 2019) approach, which is a derivative of the well‐known reduced representation method, ddRADseq (Peterson et al., 2012). 3RAD enhances the adapter ligation efficiency by employing a third restriction enzyme to cleave adapter dimers, thus preventing chimera formation and improving the final amplicon yield. For the library preparation, we followed the suggested protocol by Bayona‐Vásquez et al. (2019) with a few modifications. Three enzymes, *Eco*RI‐HF (common cut), *Xba*I (rare cut), and *Nhe*I (dimer cleaving) were used for enzyme digestion (all enzymes from Thermo Fisher Scientific). We administered the clean‐up of the PCR products using a 1:1.8 mixture of AmPure beads, followed by washing with 70% ethanol. After adapter ligation, clean‐up, and amplifications, we pooled the library and selected 500‐bp fragments (±10%) by employing Pippin Prep (Sage Science, Beverly, MA). The final library was evaluated on an Agilent 2100 Bioanalyzer (Agilent Technologies, Santa Clara, CA, USA). The prepared library was sent to Macrogen Inc. (Korea) and sequenced on 2 lanes of the Illumina HiSeq X‐10 platform with 2 × 150 paired‐end reads.

#### 2.2.2 Raw data processing

The raw sequence data were first demultiplexed and trimmed in Stacks ver. 2.41 (Rochette et al., 2019). We used the process_radtag function to filter out poor‐quality reads with high error rates (threshold Phred score 10). Due to the lack of a reference genome for *H. radicata*, the de novo strategy was applied for the RAD loci assembly. We first assembled the catalogs with the parameter settings of ‐m 3 and ‐M 3 in the ustacks function. During the catalog assembly, a maximum of one mismatch between sample loci was allowed (‐n 1, cstacks function; Paris et al., 2017).

#### 2.2.3 Genotyping

We called SNPs using the Population software implemented in Stacks with the assembled catalogs. SNPs were called only if the loci were present in at least 80% of the samples within each population and shared by at least 12 populations (‐p 12 and ‐r 0.8). We only included the first SNP per locus (‐write‐single‐snp) to avoid linkage disequilibrium (LD). SNP loci exhibiting significant departure from Hardy–Weinberg Equilibrium (HWE, threshold = *p* < 10e‐6; Li, 2011; Hess et al., 2012) were also removed to filter loci with extreme heterozygosity, likely resulting from assembly errors, using Plink ver. 1.9 (Purcell et al., 2007). Finally, we further purged the genotypes with more than 30% missing calls and SNP loci with a minor allele frequency of ≤0.05 from the dataset using Tassel 5.0 (Glaubitz et al., 2014).

### 2.3 Genetic diversity and population structure

#### 2.3.1 Genetic diversity

We computed three genetic diversity parameters, expected heterozygosity (He), observed heterozygosity (Ho), and number of alleles (Na) in GENALEX 6.5 (Peakall & Smouse, 2012). Given the variation in the number of individuals collected across populations sampled in our dataset, Na was adjusted by rarefaction curves to account for unequal sample sizes (Table 1; Kalinowski, 2004) in HP‐Rare (Kalinowski, 2005). We estimated pairwise population differentiation (*F*_ST_) among 22 populations using Arlequin 3.5, with 1000 permutations, to assess the statistical robustness of each *F*_ST_ value (Excoffier & Lischer, 2010). To examine whether population differentiation was significantly correlated with geographic distance, we conducted a Mantel test. We performed the Mantel with log‐transformed Euclidean distances as predictors on linearized *F*_ST_ [*F*_ST_ = *F*_ST_/(1 − F_ST_)] in GENALEX (Rousset, 1997).

#### 2.3.2 Population structure

Principal coordinate analysis (PCoA) and Bayesian model‐based assignment tests were used to find genomic clusters among the 22 populations. We performed PCoA based on Nei's genetic distance computed from 283 genotypes in GENALEX. We inferred the number of randomly mating clusters (K) using fastSTRUCTURE, an alternative of the widely used STRUCTURE for a large size data such as genome‐wide SNPs (Pritchard et al., 2000; Raj et al., 2014). We ran fastSTRUCTURE using the setting “logistic” due to its flexibility on population size variation and population structure (Raj et al., 2014). Values of K ranging from 1 to 12 were tested, and each K run was repeated 20 times for cross‐validation. We determine the optimal K with the function, “chooseK” implemented in fastSTRUCTURE. To visualize the expected admixture proportion inferred for the optimal K, we used a function, “distruct.py” in fastSTRUCTURE.

### 2.4 Inferring demographic history

Inferring invasion history with model‐based methods outperforms the conventional tree‐based methods, yet it has computational challenges, particularly for a large number of populations (Estoup & Guillemaud, 2010). Accordingly, we employed three different approaches to infer the colonizing history of *H. radicata*. First, demographic changes, specifically changes in effective population sizes, for all 22 populations were inferred with a simulation‐based approach. We then employed a tree‐based method to infer the history of divergence at the regional population level. Last, to reconstruct the large‐scale invasion history of *H. radicata*, the ABC model‐based approach was used. For the ABC approach, three re‐organized clusters were used to reduce the computational challenges.

#### 2.4.1 Inferring population‐level demographic changes with a simulation approach

We first estimated contemporary effective population sizes (Ne) and assessed population bottlenecks with a simulation‐based approach. The contemporary effective population sizes of 22 collected populations were estimated using the LDNe method (Waples & Do, 2008) implemented in NeEstimator (Do et al., 2014). When estimating Ne, missing data can bias the results, therefore, we pruned all SNPs with missing sequences, resulting in a final SNP dataset of 1062 SNPs. We chose the LDNe method due to its reduced susceptibility to total genetic variability (given that expected He could be highly variable in invasive species; Do et al., 2014). The threshold of 0.05 (minimum allele frequency) was applied to avoid excessive loss of loci and infinite estimates of Ne. To account for unequal sample sizes, we employed rarefaction and adjusted Ne values by the smallest sample size (=9 for our population samples) using a function implemented in NeEstimator.

**FIGURE 3:**
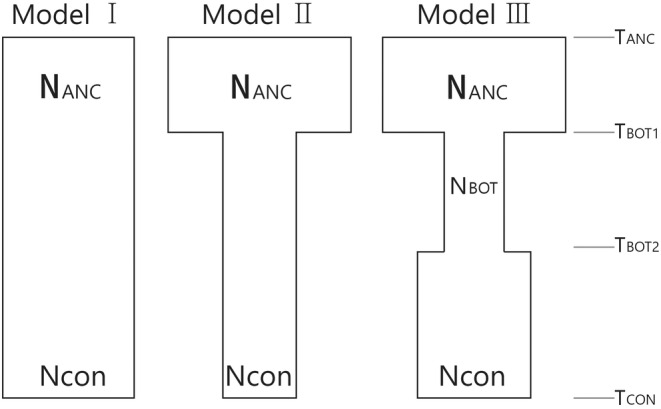
Demographic models for 22 sampled *Hypochaeris radicata* populations were tested in the simulation analysis with FASTSIMCOAL2. Included parameters: *N*_ANC_, ancient effective population size; *N*_CON_, contemporary effective population size; *T*_BOT_, time of population bottleneck initiated; *T*_ENDBOT_, time of population bottleneck ended.

We then employed a simulation‐based method implemented in FASTSIMCOAL2 (Excoffier et al., 2013) and developed demographic models to assess the population decline. To reduce the model complexity, only three simple models of a single population were tested with Ne fixed at the current time (*t*_0_). The model details are as follows (refers to Figure 3): (1) null model with constant population size, (2) population bottleneck model with two parameters – time of the bottleneck and size of the ancient population, and (3) population bottleneck and rebound model with four parameters – time of the bottleneck, time of population bottleneck end, size of the ancient population, and size of the bottlenecked population. In each demographic model, we used a mutation rate of 7 × 10^−9^ estimated from *Arabidopsis thaliana* (Krasovec et al., 2018). We generated folded site frequency spectra (SFS) of SNP loci, which were called separately for the 22 local populations to avoid missing sequence data. For composite likelihood computation, we ran 400,000 simulations and 80 ECM (Expectation Conditional Maximization) cycles repeating the run 100 times for each demographic model with a stopping criterion of 0.001 (Excoffier et al., 2013; Excoffier & Foll, 2011). We selected the best run for each demographic model using the maximum likelihood and then calculated the AIC scores for the best run of the three demographic models. AIC values were compared to choose the best demographic model for each population. For the credibility assessment of the point estimates of the demographic parameters, we estimated confidence intervals by bootstrapping. We generated 50 pseudo‐replicated SFS data from the observed SFS of each population. We then repeated the FASTSIMCOAL run on the replicated data with the best demographic model using the same conditions as in the original run. Confidence intervals were calculated based on the parameter estimates from the best run of each replicated SFS in R 4.2.2 (R Core Team, 2022). We also mapped Ne and He to visualize spatial patterns across the distribution range using the kriging function in ARCGIS v. 10.8.2 (ESRI, Redlands, CA, USA).

#### 2.4.2 Inferring history of population differentiation with a tree‐based approach

For the tree‐based method, we conducted a polymorphism‐aware phylogenetic model (PoMo) implemented in IQ‐TREE (Nguyen et al., 2015). The PoMo tree‐building algorithm is well suited for scenarios involving incomplete lineage sorting because it uses population site frequency data (De Maio et al., 2015). We calculated allele site frequency for each population using a Python script, cflib (counts file library) implemented in IQ‐TREE. The best‐fitting model was determined with a function ‐m TEST, followed by subsequent analysis (TVM + F + G4) on the best‐fitting model allowing for excess polymorphism (+P). For tree searching, we used settings designed for the short sequences, including ‐pers 0.2 (perturbation strength), ‐nstop 500 (number of stop iteration). Node supports were computed with 1000 bootstrap replicates. We visualized and adjusted the tree in FigTree (Rambaut, 2017).

#### 2.4.3 Inferring demographic history with ABC approach: divergence

**FIGURE 4:**
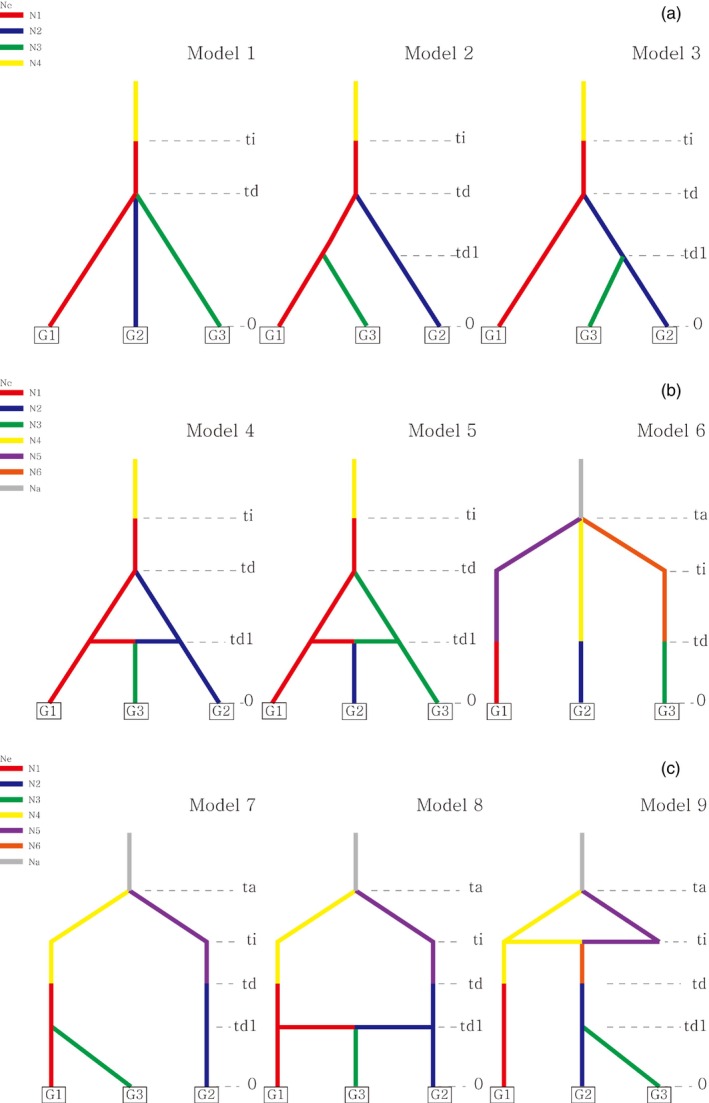
Visual representation of 9 scenarios for *Hypochaeris radicata* invasion competed in the ABC analysis. Three sampled populations that were redefined and three unsampled ghost populations were considered in the scenarios. Scenarios 1 to 5 assumed single introductions and subsequent divergence, whereas scenarios 6 to 9 infer repeated introductions from varying genetic sources. Acronyms presented in the figure: G1, G2, and G3 refer to pop 1, pop2 and pop 3 of the assigned groups in Table 1 respectively, ti, time of introduction; ta, time of divergence for the ancestral population; td, time of divergence after the introduction; td1, time of the most recent divergence; Ne, effective population size; N1 ~ N3, effective population sizes of pop1, pop2, and pop3, respectively; N4, effective population size of the introduced population; N5 ~ N6, effective population sizes of unsampled populations; Na, effective population size of the ancestral population.

To infer the introduction and divergence history of *H. radicata* invasion in Korea, we generated likely scenarios and completed them using the Approximate Bayesian Computation (ABC) approach (Beaumont et al., 2002). ABC computes posterior probabilities of different scenarios based on similarity between observed data and a large number of simulated datasets. We used DIYABC Random Forest (DIYABC–RF) ver. 1.0 (Collin et al., 2021) for the ABC computation. Although ABC can take any number of populations theoretically, dimensionality problems (please see box 2 in Estoup & Guillemaud, 2010) greatly limit the number of populations that can be used in actual analyses (Beaumont et al., 2002). To avoid the dimensionality issue, we redefined the populations into three clusters (population 1–3) based on the clustering patterns observed in fastSTRUCTURE results (see the assigned cluster for each population presented in Table 1). Eight populations with high‐admixture proportion were excluded from the analysis (Table 1). Additionally, we made up three “ghost populations” (population 4–6) to account for the unsampled genetic sources that might have been introduced to Korea from the native area (Cornuet et al., 2010). Considering the genetic clustering, distribution pattern, and prior information of *H. radicata* invasion in Korea, nine invasion scenarios were generated (Figure 4). The first three scenarios (1–3) hypothesized a single‐source introduction from the native region with instantaneous divergence (scenario 1) or subsequent divergence from the original founding population (scenario 2 & 3). An admixture event between two diverged populations from the original founding population was incorporated for scenarios 4 and 5. For scenario 6, we hypothesized that the three specified populations all independently diverged from varying genetic sources sampled in the native region through multiple introductions. The last three scenarios (7–8) incorporated multiple introductions with admixture events before (scenario 9) or after introduction (scenario 8), with subsequent divergence in scenario 7.

We generated a random forest model with 500 trees and a training data set containing summary statistics estimated in 300,000 simulated data sets for each scenario. The prior distribution of parameters was set as uniform, and the priors for the effective population sizes were set to 100–100,000. Priors for the divergence times were set in a specific order (ti > td > td1, between 10 and 60), except for ta, the divergence time in the native region (101 < ta < 10,000). We selected the best‐supported scenario based on the classification votes, the number of cases where the scenario is chosen in a forest of 1000 trees and the posterior probability (Pudlo et al., 2016) calculated in a random forest algorithm implemented in DIYABC–RF. For checking the robustness of the selected scenario, global prior errors and the mean misclassification error rate were estimated with the out‐of‐bag (out‐of‐bootstrap, OOB) training data (Breiman, 2001; Pudlo et al., 2016; Raynal et al., 2019). We also assessed the quality of prediction using visual presentation of an LDA (linear discriminant analysis) plot projecting training and observed datasets onto the first two LDA axes of the summary statistics for the selected scenario (Collin et al., 2021). The demographic parameters of the selected scenario were estimated by building a regression random forest of summary statistics with 500 trees based on 100,000 simulated data sets. The parameter precision was estimated with the global and local NMAE and estimated 95% confidence interval of the parameters.

## 3 RESULTS

### 3.1 Raw data and genetic diversity

The 3RAD library yielded ~2.2 million reads (33Gbp) with an average GC content of 37.2%. We called approximately 200,000 SNP loci from the initial SNP identification using the Population function in Stacks and subsequently pruned bad‐quality SNPs. After the quality filtering process, 3653 SNPs remained for further analysis. All three genetic diversity measures differed across populations (Table 1). The SM population showed the lowest mean expected heterozygosity (He = 0.166), while the SS population displayed the lowest mean observed heterozygosity (Ho = 0.135). In contrast, the GJ exhibited the highest heterozygosity for both the expected and observed values (He = 0.251; Ho = 0.2; Table 1). Rarefied allele numbers were highest in the HN (Na = 1.86) and lowest in the SM (Na = 1.53; Table 1). The mean *F*_*ST*_ across all population pairs was 0.096, but the values greatly varied among the individual population pairs (0.26, SM/BA – 0.03, OR/YM, PS/TH, KN/GJ; Table 2). The genetic differentiation was not significantly correlated with the geographic distances between population pairs as indicated by the weak correlation (*r* = 0.14) and low statistical support (*p* > 0.1) observed in the Mantel test (Figure S1).

**TABLE 2:** Mean pairwise *F*_ST_ values computed from 3563 SNPs among 22 *Hypochaeris radicata* populations in Korea.

	JM	JJ	YM	PS	BM	SS	KN	GS	AM	OR	TH	GI	HP	GJ	HN	MN	BA	DG	YJ	SM	KR	YA
JM	0.00																					
JJ	0.17	0.00																				
YM	0.13	0.13	0.00																			
PS	0.13	0.13	0.08	0.00																		
BM	0.13	0.12	0.07	0.07	0.00																	
SS	0.13	0.13	0.08	0.07	0.07	0.00																
KN	0.13	0.13	0.08	0.07	0.07	0.07	0.00															
GS	0.09	0.11	0.06	0.06	0.05	0.06	0.06	0.00														
AM	0.12	0.09	0.05	0.06	0.06	0.06	0.06	0.04	0.00													
OR	0.11	0.11	0.03	0.06	0.05	0.06	0.06	0.04	0.04	0.00												
TH	0.12	0.12	0.06	0.03	0.06	0.06	0.06	0.05	0.05	0.04	0.00											
GI	0.15	0.14	0.10	0.09	0.07	0.09	0.09	0.07	0.08	0.07	0.07	0.00										
HP	0.11	0.11	0.06	0.05	0.04	0.03	0.05	0.04	0.04	0.04	0.04	0.07	0.00									
GJ	0.12	0.11	0.06	0.06	0.06	0.05	0.03	0.04	0.05	0.04	0.04	0.07	0.04	0.00								
HN	0.22	0.22	0.17	0.16	0.17	0.16	0.16	0.15	0.15	0.15	0.14	0.18	0.14	0.14	0.00							
MN	0.17	0.17	0.11	0.10	0.10	0.11	0.11	0.09	0.10	0.09	0.09	0.12	0.09	0.09	0.19	0.00						
BA	0.24	0.24	0.19	0.18	0.18	0.18	0.18	0.16	0.17	0.16	0.16	0.19	0.16	0.16	0.23	0.21	0.00					
DG	0.16	0.16	0.11	0.09	0.10	0.10	0.10	0.09	0.09	0.09	0.08	0.12	0.08	0.08	0.17	0.13	0.19	0.00				
YJ	0.14	0.13	0.08	0.08	0.08	0.08	0.08	0.07	0.07	0.07	0.06	0.10	0.06	0.06	0.15	0.11	0.18	0.10	0.00			
SM	0.21	0.20	0.15	0.14	0.14	0.14	0.14	0.13	0.13	0.13	0.13	0.16	0.12	0.12	0.23	0.17	0.26	0.16	0.14	0.00		
KR	0.13	0.13	0.08	0.07	0.07	0.07	0.07	0.06	0.06	0.06	0.06	0.09	0.06	0.06	0.16	0.10	0.18	0.10	0.08	0.13	0.00	
YA	0.15	0.15	0.10	0.09	0.09	0.09	0.09	0.08	0.08	0.08	0.08	0.11	0.07	0.07	0.18	0.12	0.20	0.11	0.09	0.16	0.09	0.00

*Note*: See Table 1 for the population acronyms. All values are statistically significant at *p* < 0.01.

### 3.2 Population structure

**FIGURE 5:**
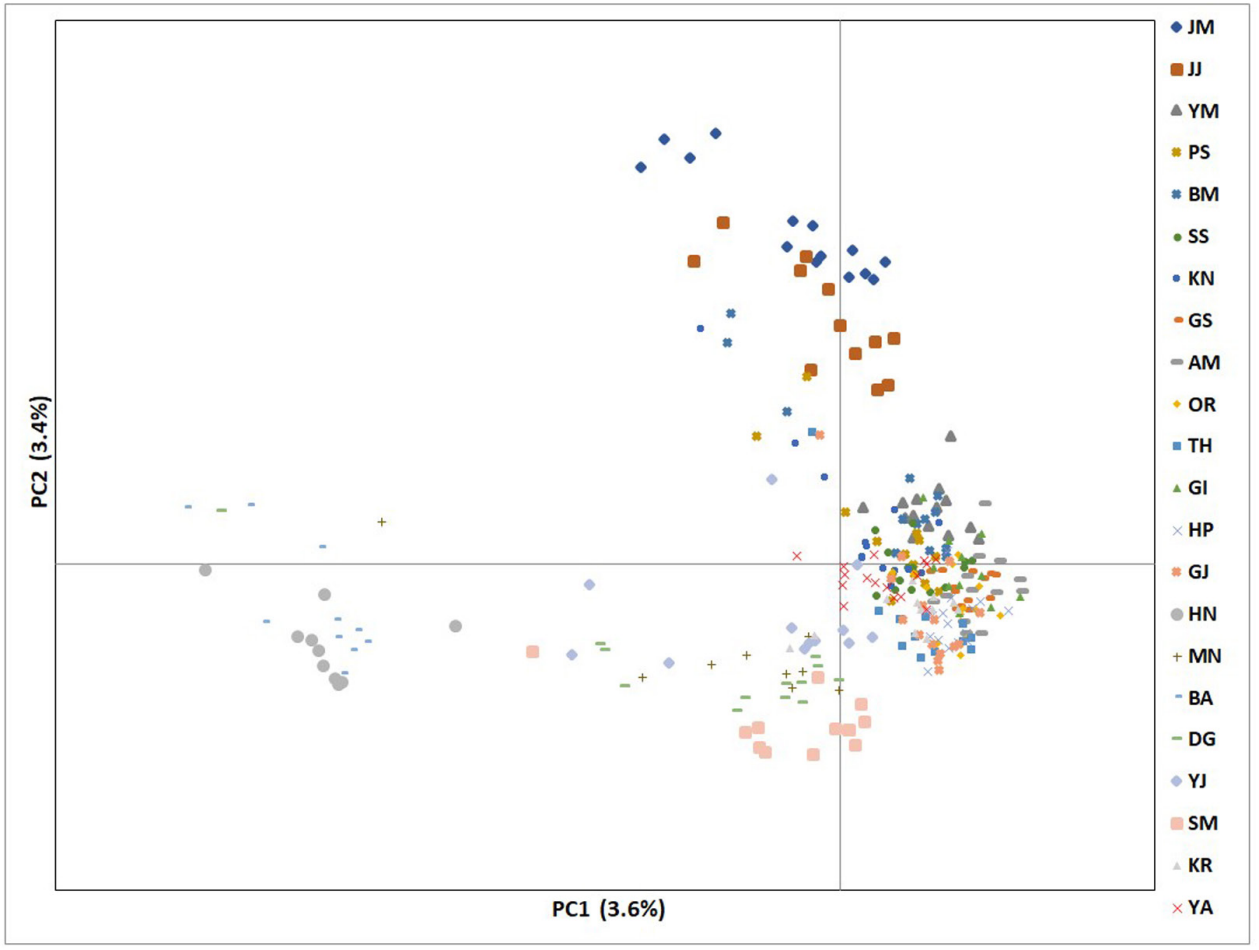
Principle coordinate analysis of 22 *Hypochaeris radicata* populations sampled in Korea.

**FIGURE 6:**
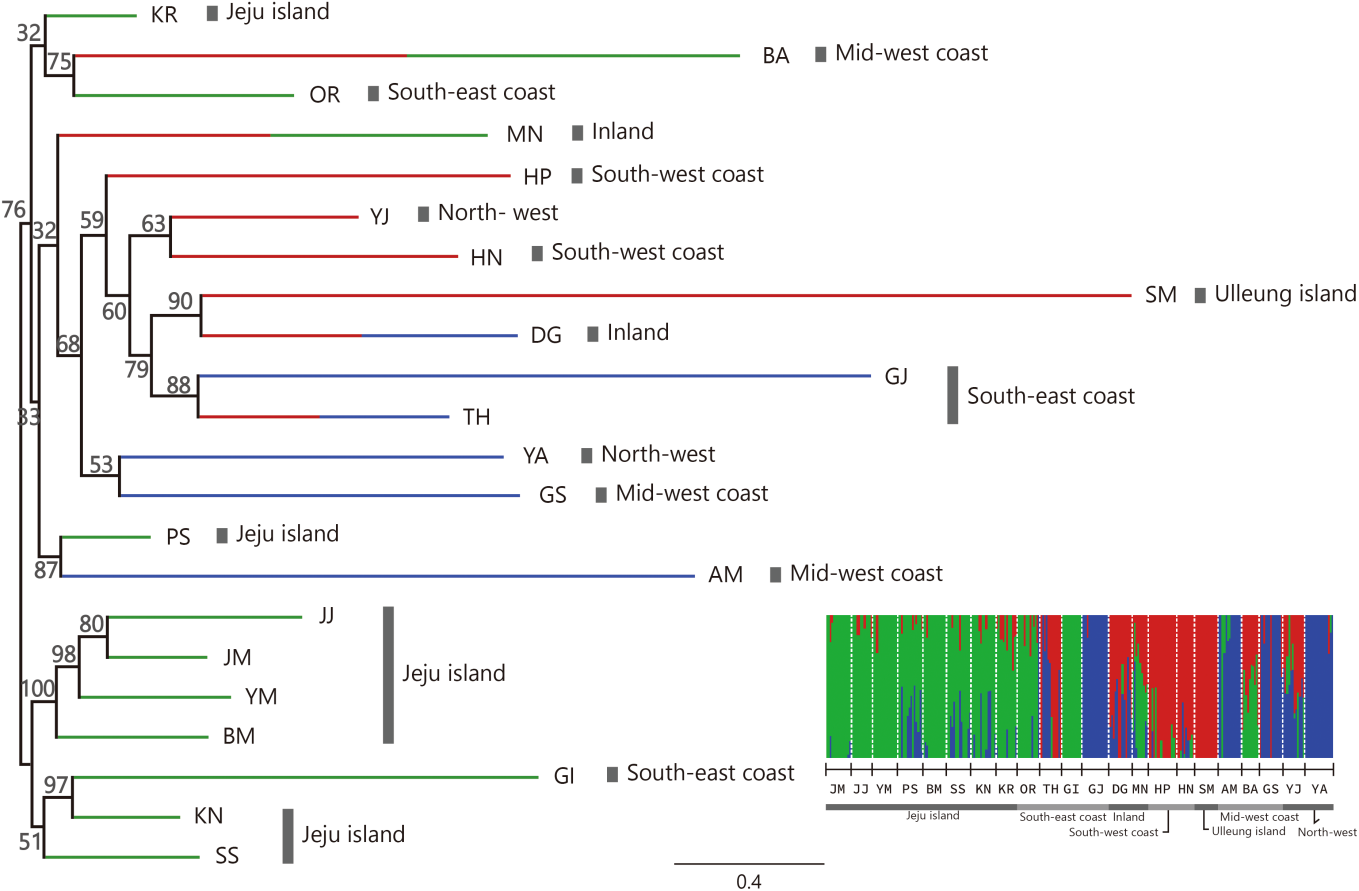
Population‐level phylogeny of 22 *Hypochaeris radicata* populations inferred from the polymorphism‐aware phylogenetic model implemented in IQ‐TREE. The numbers on the nodes indicate statistical support assessed from 1000 bootstrap replicates. Colors on tips represent the assignment groups estimated for each population by fastSTRUCTURE (*K* = 3). The bar plot on the right provides a visual representation of the *K* = 3 group assignments. Populations are separated by dotted vertical lines. The scale bars on the bottom of the bar plot indicate the population origin of each genotype. The bolded lines with black and grey color indicate the region to which populations belong. See Table 1 for population acronyms.

The overall clustering patterns differed between the PCoA and fastSTRUCTURE results (Figures 2, 5). The first three axes of PCoA collectively accounted for <10% of the total variation (Figure 5). In the PCoA plot, three populations from Jeju (BM, JJ, and JM) clustered together, while HN, along with BA, separated from the cluster mainly along the PC1 axis (Figure 5). The remaining 17 populations largely nested together in a cluster, with a few genotypes affiliated with the other two clusters (Figure 5). The range of optimal *K*, the number of clusters assuming HWE, inferred from fastSTRUCTURE, was 3–6 (Figures S2, S3). Of the K numbers, K3 best explains the geography and biological features of *H. radicata*. Therefore, we presented the Bayesian clustering results with K3. Overall, in K3, nearly all local populations exhibited low to high admixture proportions among the three genomic clusters. We also found discrepancies between geographic locations and shared ancestry among populations. For example, GJ and YA shared nearly identical assignment patterns despite the geographic distance between the two populations (Figure 2). Hereafter, for convenience, we divided our 22 collection sites into 7 regional groups by geographic locations and consistently used the group names (see Figure 2 for the regional groupings). In most regional groups, geographically neighboring populations did not share assignment patterns (Figure 6). However, eight populations from Jeju Island exhibited a similar assignment pattern predominantly represented by a green cluster with a low level of admixture (Figure 2). Genotype assignment patterns for GI and OR (south‐east coastal group) were affiliated with the Jeju group (see Table 1 for population acronyms). Most genotypes of the southwest coastal group (HP and HN) primarily assigned to a red cluster, whereas midwest coastal genotypes (GS and AM) were predominantly assigned to a blue cluster (Figure 2).

### 3.3 Population‐level demographic changes

**FIGURE 7:**
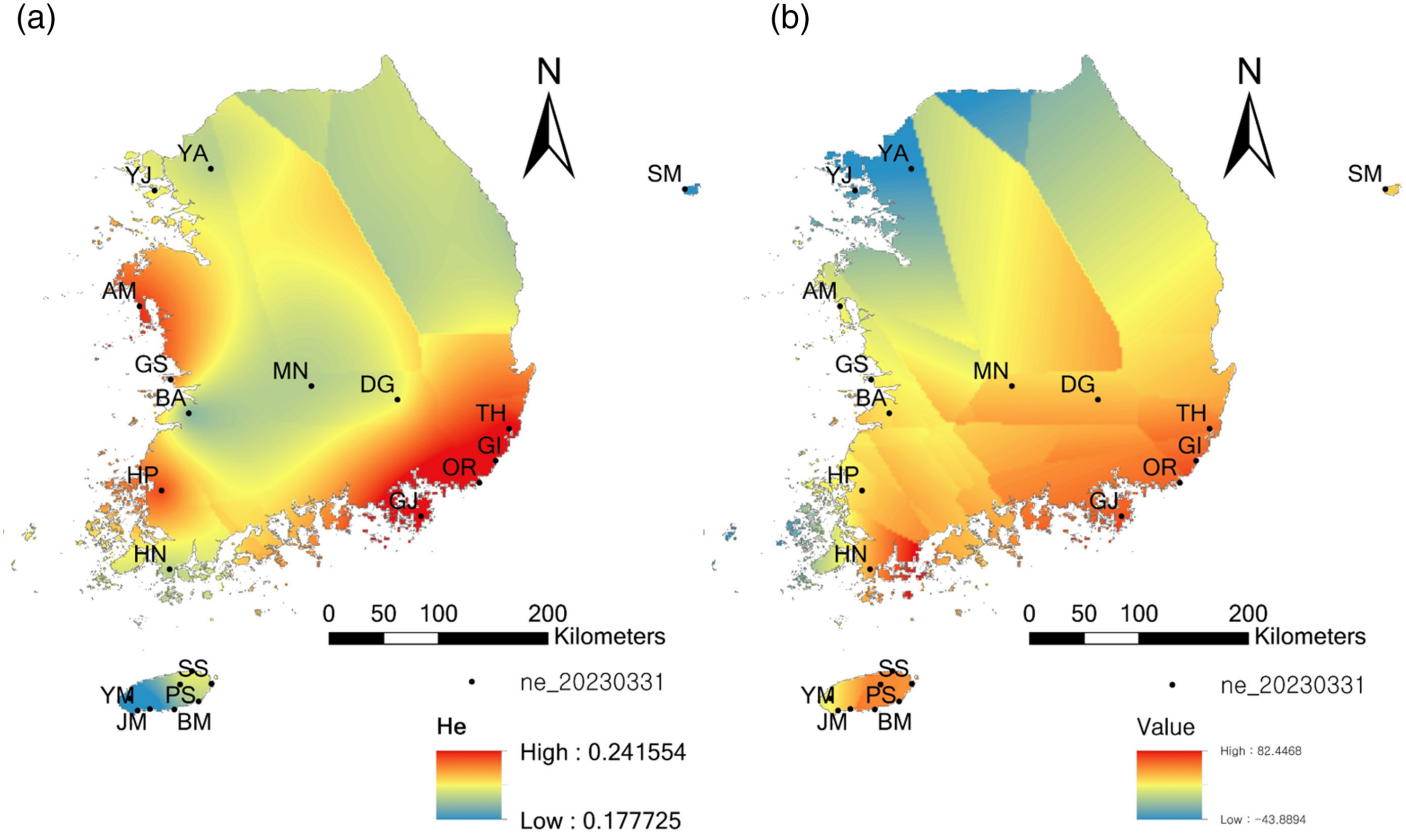
Spatial interpolation (Kriging) of 22 *Hypochaeris radicata* populations sampled in Korea. (a) mean genetic variation (He) over 3563 SNPs, and (b) contemporary effective populations (Ne) computed from NeEstimator.

To explore demographic changes within each local population, we computed contemporary and past effective population sizes. The contemporary effective population sizes, estimated from LDNe ranged from 11 (YJ) to 160 (HN), and all Ne values computed were statistically robust, as indicated by the tight range of confidence intervals (Table 3). We then interpolated Ne and He to test if there were geographic patterns of values using Kriging (Figure 7). The interpolated Ne and He displayed rather distinct patterns. For instance, the midwest coastal population, AM, harbored fairly high genetic diversity but a low effective population size. Effective population sizes were high along the southern coastal area, including the southern island of Jeju, while genetic diversity was higher in southern and western coastal populations, albeit not as prominent in southern Jeju (Figure 7).

**TABLE 3:** Parameter values of the selected demographic models inferred by coalescent simulations in FASTSIMCOAL2 for each sampled population.

Pop	Selected model	Number of loci	*N*	Ne	NANC	NBOT	TBOT	TENDBOT
AM	3	962	13	14.9 [14.3, 15.6]	1384 [1304.8, 1469.1]	11 [10.5, 11.4]	11 [10.9, 11.6]	21 [20.9, 21.6]
BA	3	645	11	17.7 [16.3, 19.2]	389 [388.7, 483.3]	21 [20.8, 62.8]	10 [6.2, 10.8]	20 [20.1, 23.6]
BM	3	898	13	42.8 [37.9, 49]	1325 [1347.1, 1366.0]	35 [30.8, 35.5]	10 [9.8, 11.2]	20 [21.4, 26.2]
DG	3	792	13	13.4 [12.7, 14.1]	1123 [1102.1, 1559.8]	24 [23.9, 26.0]	10 [8.5, 9.8]	20 [18.7, 25.4]
GI	3	906	11	45 [40.3, 50.9]	3780 [3179.0, 3201.1]	36 [27.1, 34.1]	34 [29.9, 33.2]	44 [39.6, 43.2]
GJ	3	561	15	21.8 [20.9, 22.8]	1634 [1636.1, 1717.9]	28 [22.3, 28.1]	11 [9.9, 13.1]	21 [19.1, 22.4]
GS	3	1004	13	22.9 [21.6, 24.3]	1985 [1190.3, 1691.7]	69 [60.0, 72.7]	15 [12.4, 15.7]	25 [25.3, 27.9]
HN	3	548	12	160.3 [111.2282.8]	1766 [1620.2, 1729.1]	77 [70.8, 81.8]	30 [21.3, 24.6]	40 [38.1, 41.0]
HP	3	554	16	56.1 [51.8, 61.1]	48 [44.2, 62.6]	10 [6.7, 12.2]	10 [9.1, 10.2]	20 [19.8, 31.7]
JJ	3	799	12	19.5 [18.1, 21.2]	1599 [1528.8, 1594.2]	14 [15.4, 18.2]	10 [9.54, 11.9]	20 [16.8, 19.2]
JM	3	1443	14	31.3 [28.3, 35]	2375 [2373.8, 2395.8]	56 [55.3, 66.7]	10 [11.2, 12.6]	20 [20.8, 22.8]
KN	3	818	14	15.2 [14.5, 16]	1561 [1755.6, 1886.3]	25 [16.1, 23.5]	10 [8.8, 12.6]	20 [16.7, 19.3]
KR	3	1013	12	66.8 [57.1, 80.4]	3817 [3776.3, 3840.3]	54 [48.0, 60.7]	19 [12.2, 17.1]	29 [26.8, 29.0]
MN	3	935	10	39.4 [34.3, 46.1]	231 [236.1, 309.3]	49 [47.8, 61.6]	18 [16.7, 18.6]	28 [24.8, 29.7]
OR	2	994	13	59 [52.5, 67.3]	15 [15.5, 16.5]	ㅡ	ㅡ	61 [55.2, 68.1]
PS	3	1062	14	108.3 [86.6143.7]	5033 [5037.8, 6898.8]	220 [177.3, 212.5]	34 [34.4, 37.2]	44 [37.1, 39.6]
SM	3	864	13	16.4 [15.2, 17.8]	1093 [979.4, 1111.8]	25 [24.5, 26.6]	36 [25.8, 40.0]	46 [38.5, 43.8]
SS	3	911	14	82 [68.5101.8]	7128 [6217.8, 7160.8]	62 [59.7, 71.9]	33 [33.8, 36.8]	43 [39.9, 51.9]
TH	3	986	12	138.3 [105.6198.6]	3730 [3380.2, 3787.3]	37 [27.7, 40.4]	10 [8.1, 10.8]	20 [16.0, 28.1]
YA	3	961	16	29.3 [27.2, 31.7]	1249 [1290.8, 1721.9]	25 [24.9, 27.6]	10 [8.9, 10.6]	20 [20.1, 22.3]
YJ	3	857	12	11 [10.5, 11.6]	1022 [1034.5, 1168.1]	12 [10.3, 12.0]	10 [9.5, 11.2]	20 [14.2, 21.0]
YM	3	1388	14	41.4 [37.5, 46.2]	2929 [2918.7, 3033.7]	56 [52.2, 57.8]	10 [9.2, 13.7]	20 [20.6, 21.7]

*Note*: Number of loci are the number of SNP loci used to compute the folded SFS. The SNP loci were called separately for the 22 local populations to avoid missing sequence data. See Table 1 for the population acronyms and Figure 3 for the selected demographic models. Time was estimated backward from the present. Numbers in the brackets are the confidence intervals at the 95% level.

Abbreviations: NANC, effective population size of initial populations before bottleneck; NBOT, effective size of bottlenecked populations; Pop, population; TBOT, time of population decline ended; TENDBOT, time of population decline initiated.

Of the three demographic models (Figure 3), model 3, which posits a population bottleneck followed by recent expansion, had the strongest statistical support based on AIC values in most populations except for one population (model 2 for OR; Table S1). The four‐point estimates (NANC, NBOT, TBOT, and TENDBOT) differed largely among populations (Table 3). The mean values for NANC (effective size of initial populations before bottleneck) and NBOT (effective size of bottlenecked populations) were 588 and 16, respectively. Several populations in Jeju (PS, KR, SS, and JM) showed the most drastic population decline followed by gradual growth (Table 3). Additionally, MN and BA, exhibited a modest population decline. The onset of population decline commenced approximately 20 years ago for several populations (see Table 1 for TENDBOT), with local populations subsequently starting to re‐expand about 15 years ago (see Table 1 for TBOT). Multiple populations experienced a fairly recent population decline (TENDBOT = 10) and rebounded within ~5 years (TBOT = 5; Table 3). However, point estimate values in some populations did not align with the chosen demographic history models and showed statistical credibility significantly above the estimated values (Table 3).

### 3.4 History of spreading populations

We found a geographically complex pattern of clades in the polymorphism‐aware phylogenetic tree (Figure 6). Overall, the tree topology did not reflect geographic regions and showed similar cluster patterns to the fastSTRUCTURE results. Many populations formed clades with populations from distant regions except for Jeju populations. Six out of eight Jeju populations clustered together with one inland population (GI), whereas the remaining two Jeju populations (KR and PS) were nested within a clade with three different coastal populations (Figure 6). Populations with genotypes primarily assigned to the red cluster in the fastSTRUCTURE results (HP, HN, SM, and YJ) were nested together in a clade (Figure 6). Based on the branch length, Ulleung island (SM), a volcanic island situated far east of the Korean peninsula, was the most diverged population and was nested with DG, an inland population (Figure 6).

### 3.5 History of introduction

We selected the best‐supported introduction history using the scenario choice prediction following Collin et al. (2021). In the projection of our observed data on the first two LDA axes, the chosen scenario was discriminated against the remaining 8 scenarios (Figure S4). RF classification votes (419 out of 500) and posterior probability (~0.9) were highest for the scenario 6 (Figure 4), indicating that the scenario 6 was the best suited demographic scenario (Table 4). However, the global (0.4) and local error rates (0.1) from the out‐of‐bag procedure were relatively high, suggesting only moderate robustness of our scenario choice. The most informative feature vectors for the scenario choice included *F*_ST_‐related statistics for each population, three‐sample f‐statics, and the corresponding three‐sample coefficients of admixture (AML statistics; Figure S5). We estimated the parameter values of the selected scenario with PLS axes included to improve prediction accuracy (Collin et al., 2021). On average, the mean parameters showed smaller error rates for both global and local NMAE estimation (Table S1). The time of introduction (mean ti) and subsequent divergence time (mean td) were 47.7 and 27.8, respectively (Table S1). The two time‐related estimates, ta and ti, were statistically much more robust than the remaining 7 parameters, evidenced by the tighter 90% CI ranges and lower error rates (Table S1). The statistical support for the estimated effective population sizes of three redefined groups in the ABC analysis were weak, showing large error rates and wide 90% CI range (Table S1).

**TABLE 4:** Summary of ABC scenario selection for *Hypochaeris radicata*.

Scenarios	1	2	3	4	5	6	7	8	9
Votes	0	3	1	0	4	419	7	32	34
Posterior probability						0.894			
Global error						0.409			
Local error						0.106			

*Note*: Posterior probability and both global and local errors are provided for the selected scenario. See Figure 4 for the detailed illustrations of the 9 scenarios.

## 4 DISCUSSION

While *Hypochaeris radicata*, a cosmopolitan species occurring on nearly all continents, has been extensively explored in Western Europe, its invasion dynamics beyond Europe remain largely unknown Here, our study offers the first population‐level genomic data investigating the invasion dynamics of the successful weed outside of Europe. Consistent with our hypotheses, the invasion of *H. radicata* in Korea can be summarized by multiple introductions, population bottlenecks, and relatively short divergence times. Our data suggest that *H. radicata* has been introduced to Korea from at least three different sources within the past half‐century. Following the introductions, the founding populations experienced reduced effective population sizes but also rapidly spread throughout the country and rebounded from contraction within a decade. Despite the demographic changes, the genetic variability of these populations remained relatively high. Collectively, our results demonstrate that *H. radicata* in Korea is genetically diverse throughout the region, with the potential to further expand its ranges from diverse genetic sources.

### 4.1 Introduction history and subsequent demographic shifts

The ABC result revealed multiple introductions of genetically divergent lineages during *H. radicata* invasion in Korea. While the early observations of the plant were primarily limited to Jeju Island, suggesting it as the likely source of invasion, our ABC inference indicated that at least three genetically divergent sources were independently introduced to Korea within the last 50 years. In addition, clustering results identified three or more genetic groups in the sampled populations, with evidence of admixture between the genetic groups. The spatial clustering pattern from Bayesian clustering further suggests that *H. radicata* was introduced not only to Jeju island but also to certain parts of the mainland, rapidly spreading to other parts of the Korean peninsula, including a remote volcanic island (Ulleung island) in the East Sea.

We expected a decrease in Ne of the plant, considering the demographic bottlenecks following the relatively recent introduction history (Braasch et al., 2019; Colautti et al., 2017; Dlugosch et al., 2015; Estoup & Guillemaud, 2010). Consistent with our hypothesis, the contemporary Ne estimated (mean LD‐Ne ≈ 47) was relatively small, similar to those observed in species that have undergone demographic bottlenecks (e.g., LD‐Ne > 130 in the invasive *Euphorbia virgata* in the US, Lake et al., 2023; LD‐Ne ranging from 1 to 51 in the endangered perennial *Viola uliginosa*, Lee et al., 2020). Our simulation analysis with FASTSIMCOAL revealed that nearly all sampled populations experienced recent population bottlenecks followed by subsequent population expansion. Thus, the small contemporary Ne likely resulted from the population bottlenecks, with some populations initiating demographic re‐expansion within the last 5–18 years. However, the reduced effective population sizes were not strongly associated with the genetic diversity in our data analysis. Populations that experienced severe population bottlenecks, such as GJ, GI, JM, KR, PS, and YM (as indicated by large differences between NANC and NBOT in Table 3), harbored moderate to high genetic diversity. Indeed, the correlation between log‐transformed Ne and He in our data was weak (*r* = 0.18, *p* > 0.05; Figure 7). It suggests that genetic diversity loss influenced by Ne might have been confounded by other factors, such as admixture and gene flow among populations and/or lineages.

Latitudinal gradients in Ne were evident, as shown in the Kriging result (Figure 7b). Specifically, southern coastal populations, including Jeju island, had relatively larger Ne values than those in the mid‐ or northern populations. Although only marginally significant, contemporary Ne was related to the Ne of the past (Ne at the time of introduction, NANC; *r* = 0.46, 0.1 > *p* > 0.05). Historical records suggested pastures and agricultural fields as potential introduction points (Ahn et al., 2001; Sun et al., 1992), with many agricultural farms and cattle fields located in the southern parts and Jeju Island in Korea. Jointly, the results suggest that the southern coastal area, with increased Ne, likely served as the introduction point with larger propagule sizes. This result somewhat deviated from our initial hypothesis, which suggested Jeju as the introduction point. However, our data indicated that multiple populations in the southern coastal area, including Jeju, could have been the introduction points. Supported by the multiple introductions proposed by the ABC result, our data suggested that the plant might have been initially introduced to Jeju and the southern coastal area multiple times before spreading inland towards the north.

### 4.2 Range expansion

The overall clustering pattern of the polymorphism‐aware phylogenetic model (PoMo) tree and fastSTRUCTURE Bayesian clustering results revealed similar genetic relationships among the sampled populations. However, in the PoMo tree, the support values for nearly half of the clades were less than 50, indicating low confidence levels, which was not surprising considering the recent colonization history of the species. Indirect demographic inferences with tree topologies and distances, such as those in the PoMo tree, may not be suitable for colonizing species with short divergence times (Estoup & Guillemaud, 2010). Strong population bottlenecks, which are often expected during colonization, can compensate for short divergence time and lead to higher genetic divergence (Estoup & Guillemaud, 2010), but this was not the case in our study. Notably, the PoMo tree and Bayesian clustering results were similar, and both demonstrated no relationship between the genetic clustering patterns and spatial locations. Considering that *H. radicata* is expected to have high dispersal distances (wind‐dispersed, with seeds falling ~16 km/h from the maternal plant and further carried by gusts of wind; Turkington & Aarssen, 1983), spatially neighboring populations would likely have opportunities for gene flow and shared generic variation. Instead, multiple introductions from different sources might explain differentiation between nearby populations, and this scenario was strongly supported by the ABC modeling.

### 4.3 Genetic diversity pattern

Genetic diversity loss is often expected during biological invasion (Baker, 1955; De Pedro et al., 2021; Dlugosch et al., 2015; Dlugosch & Parker, 2008a; Encinas‐Viso et al., 2022; Maebara et al., 2020). In previous genetic studies, *H. radicata* showed relatively low population differentiation under various conditions, with no significant differences in genetic diversity observed across varying habitat conditions (Mix et al., 2006; Ortiz et al., 2008). For example, analysis of gene diversity across 44 *H. radicata* populations, based on 517 AFLP fragments, reported no notable difference between native and introduced areas (Ortiz et al., 2008). Similarly, a study utilizing microsatellite data from 17 populations sampled across well‐preserved and severely fragmented habitats found no correlation between genetic diversity and population sizes (Mix et al., 2006). Our data align somewhat with these earlier findings, indicating only minor differences in within‐population genetic diversity throughout the sampled populations, despite varying habitat and demographic conditions. Therefore, although our data cannot directly be compared with the genetic diversity of native populations, a significant reduction of genetic diversity during *H. radicata* invasion in Korea seems unlikely. However, considering the differences in molecular methods used, caution should be taken when comparing our data with previous studies. Various molecular techniques can have a significant impact on genetic diversity assessment, affecting factors like polymorphism levels, reproducibility, and genomic abundance (Bachmann, 1994; Mondini et al., 2009; Nybom, 2004).

Despite the demographic bottlenecks inferred for all sampled populations during the introduction, we did not observe strong genetic bottlenecks. Gene flow, particularly in a plant species with high dispersal potential such as *H. radicata*, might contribute to homogenizing local populations and ameliorating genetic diversity loss in small and isolated populations (Ellstrand & Rieseberg, 2016; Frankham, 1996; Sork & Smouse, 2006). Additionally, the self‐incompatible nature of *H. radicata* (Turkington & Aarssen, 1983) might also help prevent strong genetic bottlenecks (Hamrick & Godt, 1996). Alternatively, admixture of alleles, formerly isolated but brought together through multiple introductions, could be another factor mitigating diversity loss (Dlugosch et al., 2015). In fact, the multiple introductions inferred from our model‐based analysis supported the admixture hypothesis. Notably, the Kriging analysis failed to show a correlation between the effective population size and genetic diversity, likely due to the admixture events. Collectively, the weakened genetic bottlenecks documented in our study, despite the history of recent colonization, can be attributed to the combined effects of these three mechanisms.

## 5 CONCLUSIONS

Multiple introductions facilitated by human assistance are one of the major components conferring invasion success (Barbosa et al., 2019; Dlugosch & Parker, 2008a; Ellstrand & Schierenbeck, 2000; Gioria et al., 2023; Keller & Taylor, 2010). Over the past few decades, *H. radicata* has rapidly colonized nearly half of the Korean peninsula, demonstrating high invasiveness. Our study suggests that admixture among divergent sources from repeated introductions has played a significant role in the successful invasion. Admixture may enhance invasion success by preserving genetic diversity against bottleneck effects and generating novel genotypes better suited to new environments (Gillis et al., 2009; Mairal et al., 2022). Given the ongoing influx of new propagules into agricultural lands, the species is likely to further expand its range unless strong regulation and close monitoring are enforced in Korea.

## CONFLICT OF INTEREST STATEMENT

The author declares no conflict of interest.

## Supporting information


Data S1:

## Data Availability

Raw DNA sequences obtained from 3‐RAD genotyping are available as GenBank accession number PRJNA961839. The SNPs matrix and SFS matrix used in this study are publicly available on Dryad at the following DOI: 10.5061/dryad.h18931zv2.
