# Supplementary material for: Multiple introductions of divergent lineages and admixture conferred the high invasiveness in a widespread weed (Hypochaeris radicata)
Source: Evol Appl. 2024 Jun 21;17(6):e13740. doi: 10.1111/eva.13740 (PMC11192970; doi:10.1111/eva.13740)
Supplement: Supplementary file 1 — Data S1: [file EVA-17-e13740-s001.docx]

Supplementary Information

Multiple introductions of divergent lineages and admixture conferred the high invasiveness in a wide spread weed (*Hypochaeris radicata*)

Soo-Rang Lee^1*^, Tae-Young Choi^1^ and Dong-Chan Son^2^

^------------------------------------------------------------------------------------------------^

^1^Department of Biology Education, College of Education, Chosun University, Gwangju 61452, South Korea

^2^Division of Forest Biodiversity and Herbarium, Korea National Arboretum, Pocheon 11186, Republic of Korea

Table S1. AIC values estimated for three demographic models of 22 sampled populations.

| Population | Model 1 | Model 2 | Model 3 |
| --- | --- | --- | --- |
| AM | 134.98 | 76.64 | 65.08 |
| BA | 97.31 | 66.15 | 50.126 |
| BM | 134.1 | 96.655 | 89.997 |
| DG | 116.04 | 89.886 | 78.16 |
| GI | 138.39 | 88.94 | 86.39 |
| GJ | 75.42 | 45.31 | 38.75 |
| GS | 152.69 | 108.78 | 105.70 |
| HN | 75.11 | 47.75 | 46.76 |
| HP | 87.31 | 83.68 | 82.63 |
| JJ | 119.48 | 98.58 | 83.65 |
| JM | 208.89 | 147.38 | 136.94 |
| KN | 117.49 | 88.53 | 81.19 |
| KR | 173.98 | 134.42 | 133.24 |
| MN | 0.48 | 0.15 | 0.11 |
| OR | 172.889 | 135.45 | 136.37 |
| PS | 166.12 | 120.69 | 120.24 |
| SM | 130.04 | 60.42 | 40.37 |
| SS | 135.97 | 93.03 | 91.92 |
| TH | 233.22 | 207.66 | 205.17 |
| YA | 137.99 | 99.67 | 94.18 |
| YJ | 140.06 | 123.72 | 116.65 |
| YM | 207.26 | 148.32 | 140.34 |


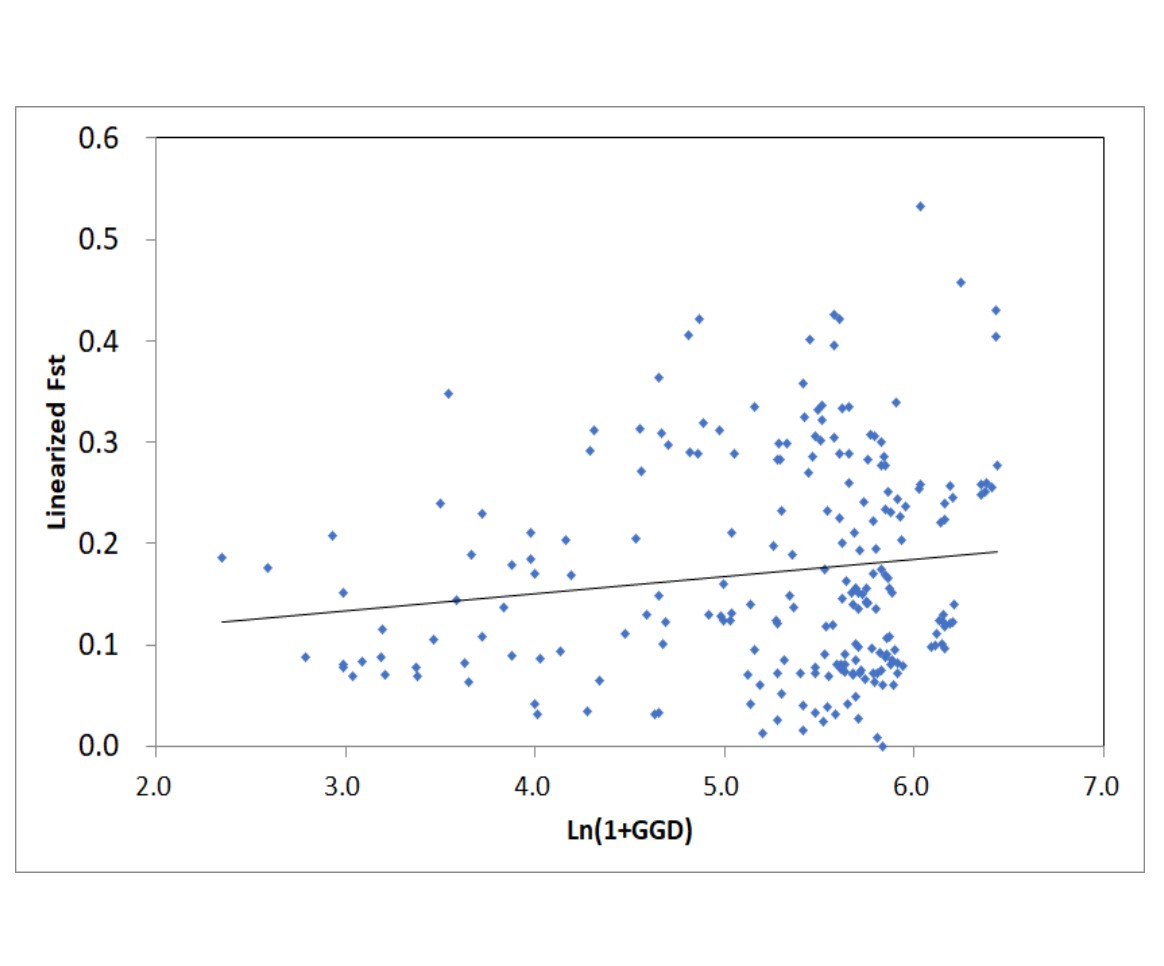
Figure S1. Plot for the result of Mantel test (r = 0.1, p > 0.1). The association between log-transformed Euclidean distance (km) and Slatkin’s linearized F_ST_ (F_ST_/(1 − F_ST_)) population pairs among 22 *Hypochaeris radicata* populations from South Korea.


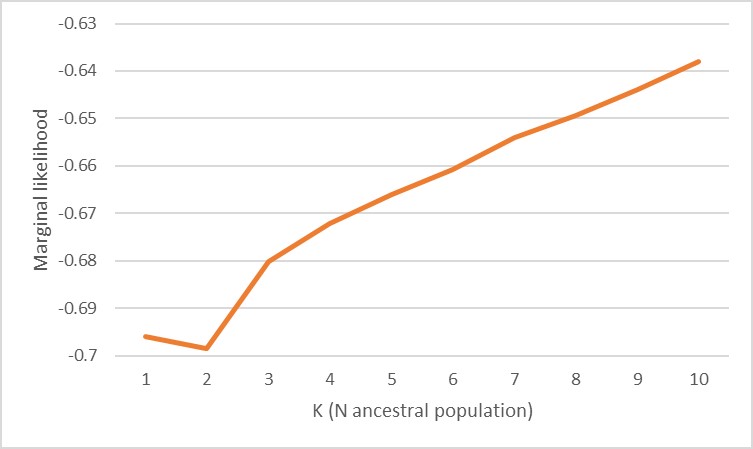


Figure S2. Plot of marginal likelihood estimated from fastSTRUCTURE for K = 1 - K = 10. The optimal K range proposed by function chooseK was 3 to 6.


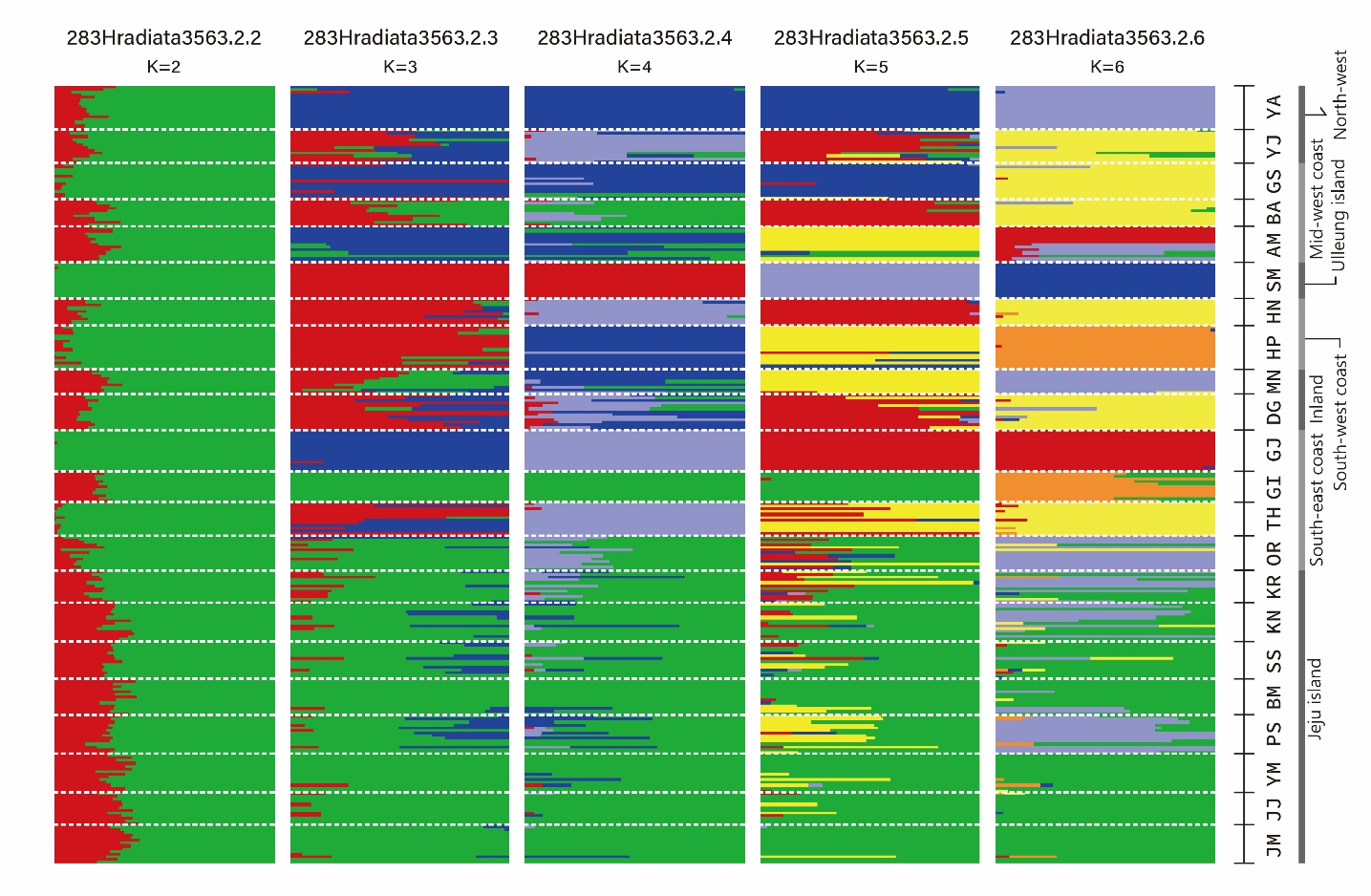


Figure S3. Bar plots of clustering assignment for K = 2 - K = 6 computed from fastSTRUCTURE using 3,563 SNPs sampled across 283 *H. radicata* genotypes. Populations are separated by dotted horizontal lines. The scale bars on the right side indicate the populations each genotype assigned and the bolded lines with black and grey color refers to the region to which populations belong. See Table 1 for population acronyms.


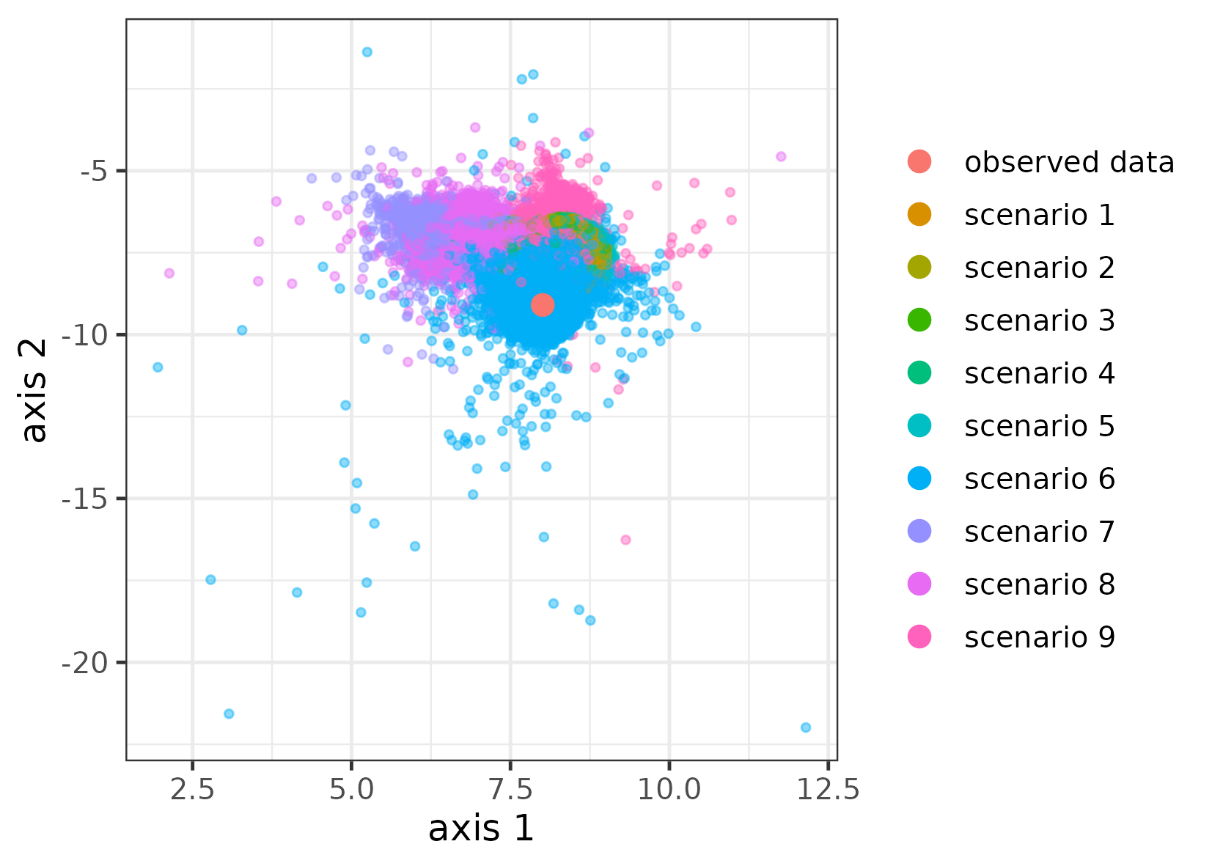


Figure S4. LDA (linear discriminant analysis) plot projecting training and observed data set on the first two LDA axes of the summary statistics for the selected scenario.


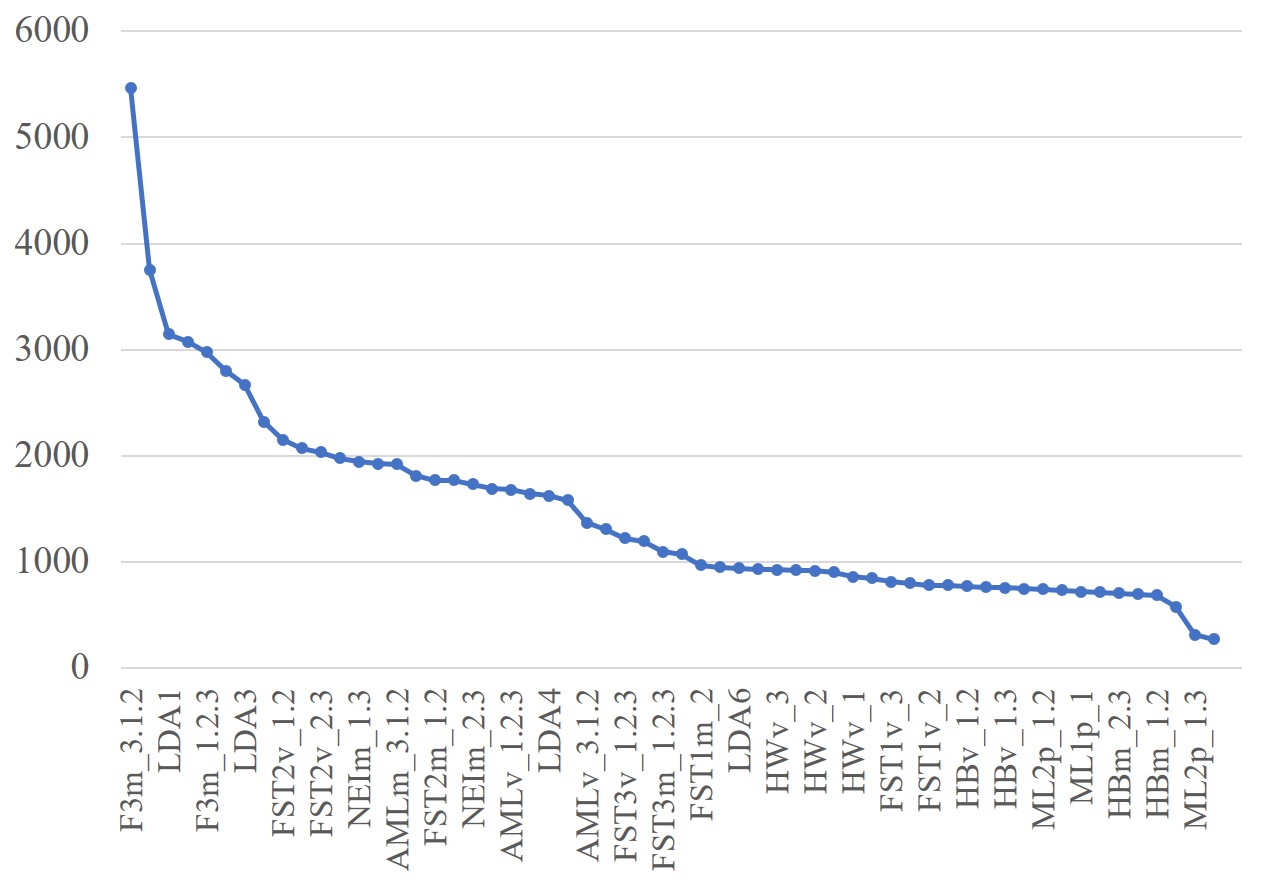


Figure S5. Contribution of 30 most informative statistics to the random forest when selecting among the 9 scenarios.
